# Primary iliac bone tuberculosis: a case report

**DOI:** 10.1099/acmi.0.000844.v3

**Published:** 2025-05-07

**Authors:** Hajar Dahou, Fataou Saley Younoussa, Imane Aragon, Salma El Aouadi, Yahya El Harras, Elmostafa Benaissa, Yassine Ben Lahlou, Abdelaali Bahadi, Jamal El Fenni, Adil Maleb, Mariama Chadli, Mostafa Elouennass

**Affiliations:** 1 Department of Clinical Bacteriology, Mohammed V Military Teaching Hospital, Faculty of Medicine and Pharmacy of Rabat, Mohammed V University, Rabat, Morocco; 2 Nephrology Departement, Mohammed V Military Teaching Hospital, Faculty of Medicine and Pharmacy of Rabat, Mohammed V University, Rabat, Morocco; 3 Radiology Departement, Mohammed V Military Teaching Hospital, Faculty of Medicine and Pharmacy of Rabat, Mohammed V University, Rabat, Morocco; 4 Laboratory of Microbiology, Mohammed VI University Hospital, Faculty of Medicine and Pharmacy (University Mohammed the first), Oujda, Morocco

**Keywords:** peritoneal dialysis, primary iliac bone tuberculosis, GeneXpert®

## Abstract

Tuberculosis (TB) remains one of the world’s leading causes of morbidity and mortality. It occurs in both pulmonary and extra-pulmonary forms. Primary iliac bone TB remains a rare clinical entity, even in endemic areas. The diagnosis of the disease can be challenging due to its similarity to other bone diseases. We report a rare case of primary iliac bone TB in a 63-year-old patient who was on peritoneal dialysis and had a medical history of hypertension and type II diabetes, which was complicated by diabetic retinopathy and diabetic kidney disease. Magnetic resonance imaging revealed osteomyelitis in the iliac bone, while real-time polymerase chain reaction using the GeneXpert^®^ system on a gluteal collection sample confirmed the diagnosis of TB. The integration of advanced molecular tools, such as GeneXpert^®^, represents significant progress, enabling rapid and accurate diagnosis of TB and facilitating early initiation of treatment.

## Data Summary

No data were generated during this research or are required for the work to be reproduced.

## Introduction

Tuberculosis (TB), caused by the *Mycobacterium tuberculosis* complex (MTBC), remains a major public health challenge and is one of the leading global causes of morbidity and mortality [1]. Morocco is classified as a country with moderate TB incidence. According to the World Health Organization (WHO) estimates from 2021, ~35,000 cases were reported, corresponding to an incidence rate of 94 per 100,000 population. Notably, the distribution of TB cases shows a relatively high proportion of extra-pulmonary forms (49%) compared to pulmonary forms (51%) [2].

While pulmonary TB is the most common manifestation [3], extra-pulmonary TB accounts for a significant proportion of cases, with skeletal involvement occurring in ~10%. The spine is the most frequently affected site, followed by other osteoarticular locations [4].

Primary TB of the iliac bone is rare, accounting for less than 1% of all cases of TB affecting the skeletal system [5]. Establishing its precise diagnosis can be difficult. Accurately diagnosing this condition can be challenging, as its symptoms often resemble those of many other diseases. We present a rare case of primary iliac bone TB in a patient undergoing peritoneal dialysis.

## Case presentation

We present the case of a 63-year-old patient originating from Khemisset, Morocco, with no recent travel history. He had a medical history of hypertension and type II diabetes complicated by diabetic retinopathy and diabetic kidney disease and had been undergoing peritoneal dialysis since 2021 via a peritoneal catheter with two isotonic and intermediate glucose-containing dialysates. He was admitted to the nephrology department for the management of an infectious syndrome characterized by a fever of 38.5 °C, chills and vomiting. He also reported a weight loss of 10 kg over the past month, accompanied by inflammatory polyarthralgia of the large joints and low back pain for 5 days, resulting in gait impairment.

Clinical examination revealed localized pain in the left iliac wing. Blood pressure of 130/70 mm Hg, weight of 82.5 kg and diuresis preserved were observed. Pleuropulmonary examination revealed SaO2 at 98%, respiratory rate at 17 cycles per minute and fine crackles at the lung bases.

Biological assessment revealed a haemoglobin level at 8.6 g dl^−1^, GMV at 84 fl, C-reactive protein (CRP) at 94 mg L^−1^ and a procalcitonin level of 2.57 ng ml^−1^. Microbiological investigations, including cytobacteriological examinations of urine and peritoneal dialysis fluid, returned sterile results. Blood cultures obtained from both aerobic and anaerobic bottles were also negative. Conventional methods for detecting MTBC, including direct examination (DE) and culture of sputum samples collected over 3 consecutive days, as well as molecular biology approaches, all yielded negative results.

**Fig. 1.**
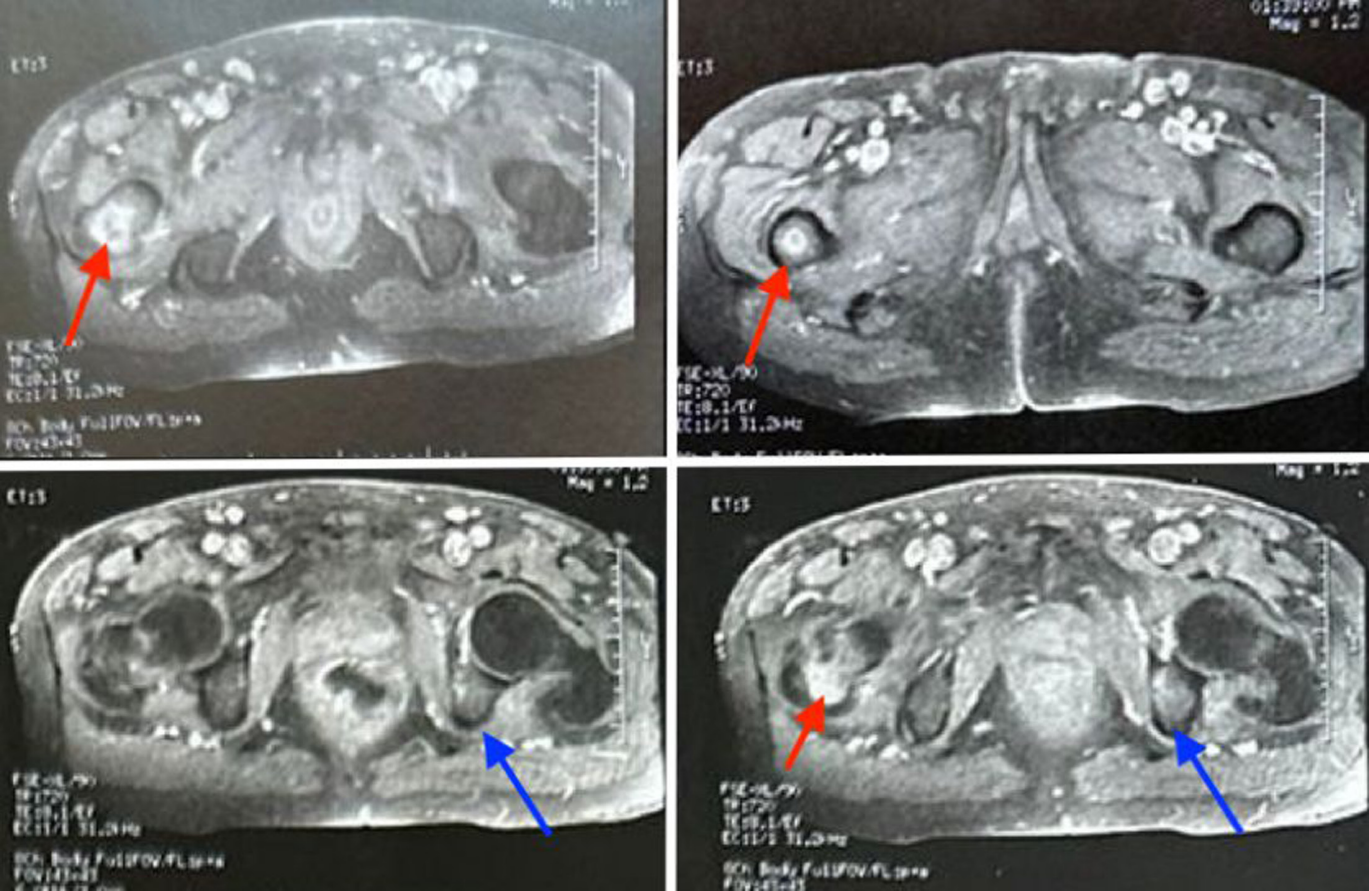
Axial pelvic T1-weighted MRI sequences after gadolinium injection show enhanced intramedullary foci. The red arrow indicates a hyperintense lesion in the right greater trochanter and metaphysis, while the blue arrow highlights a similar lesion in the left iliac bone, both demonstrating post-contrast enhancement consistent with foci of osteomyelitis.

With acute pyelonephritis and infectious peritonitis cast aside, we proceeded to radiological exams. The computed tomography (CT) of the lumbar spine showed no particular findings except for some minimal arthritic changes at the thoracolumbar level. The magnetic resonance imaging (MRI) of the pelvis revealed a focus of osteomyelitis of the right femur and a focus of osteitis of the left ilium, with extension into the adjacent muscle soft tissues and suspicion of intramuscular collections (Fig. 1).

**Fig. 2.**
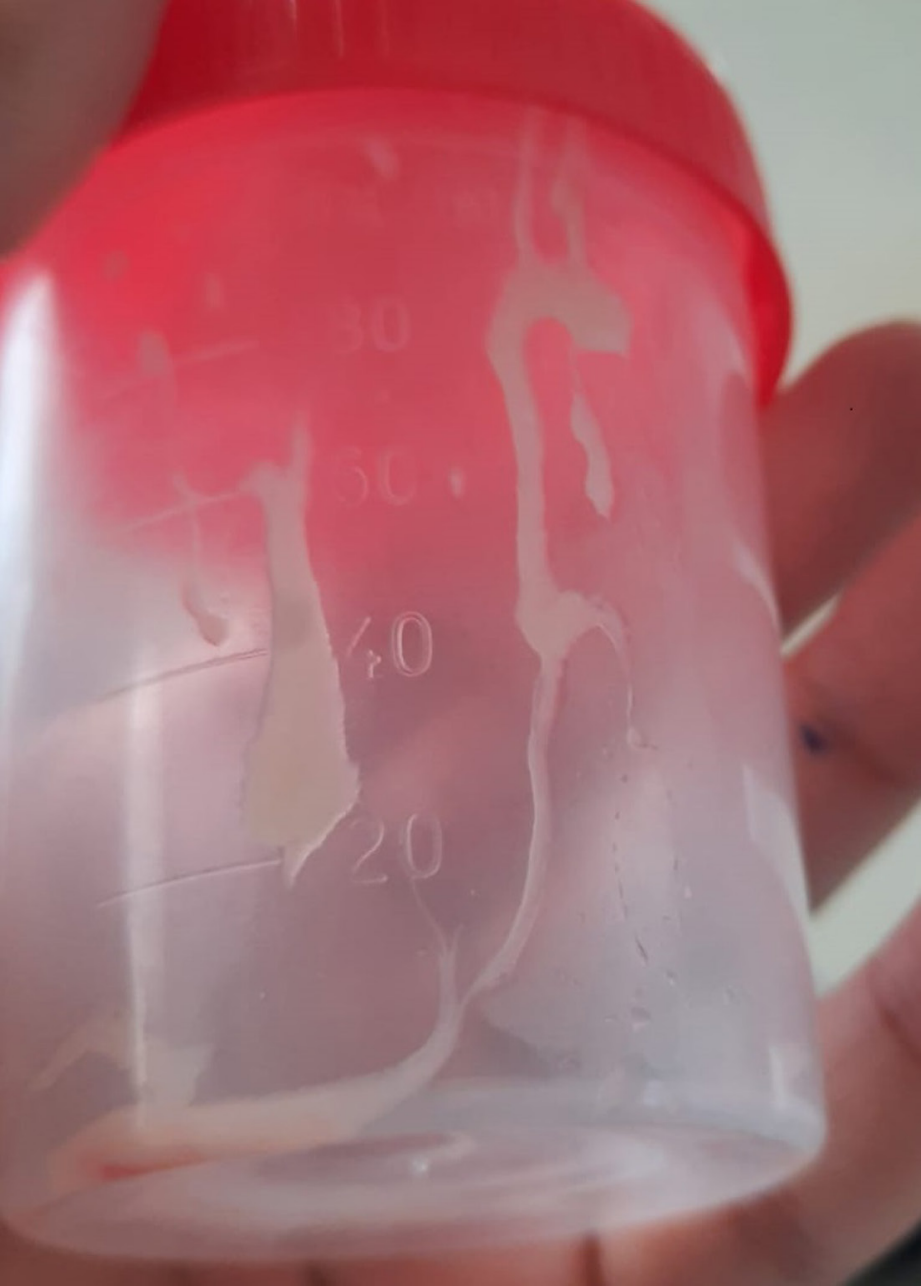
Sample obtained by puncture of the gluteal collection.

**Fig. 3.**
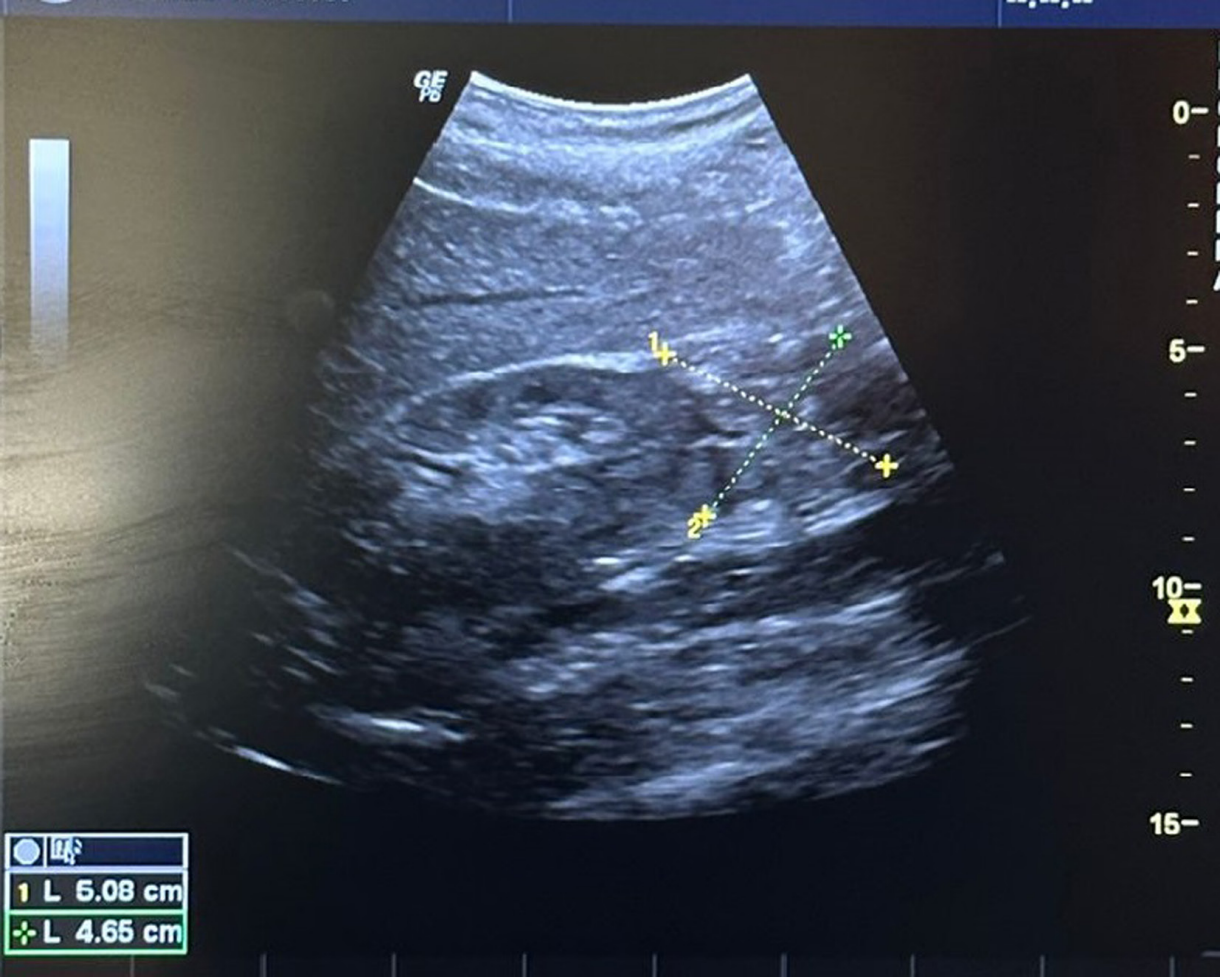
Ultrasound of the soft tissues showing a heterogeneous gluteal collection within the left gluteal muscle, measuring 5.08 cm × 4.65 cm.

Molecular biology testing using real-time polymerase chain reaction (PCR) with the GeneXpert^®^ system for MTBC was performed on a sample (Fig. 2) obtained by ultrasound-guided puncture of one of the gluteal collections (Fig. 3), which turned positive. Culture from this same sample on Lowenstein–Jensen (LJ) medium tested positive after 21 days.

The diagnosis of iliac bone TB was confirmed on the basis of radiological (MRI) and microbiological (PCR) results.

The patient was initiated on anti-TB treatment with dosages carefully adjusted to his renal function to prevent toxicity related to impaired drug elimination. The course was marked by significant clinical and biological improvement, with normalization of inflammatory parameters and good tolerance to the anti-TB treatment.

At the 3 months follow-up under anti-TB treatment, most of the clinical symptoms faded, yet the pain remained at 7/10 in intensity, impeding his two solution changes throughout the day. After accumulating 9 days without proper peritoneal dialysis, he was switched to emergency haemodialysis on a symptomatic uremic syndrome (urea level: 4.2 g l^−1^) through a jugular catheter. At a 6 month follow-up, the patient is still undergoing haemodialysis through an arteriovenous fistula, at the proper dialysis dose, and has regained his quality of life.

## Discussion

Bacterial infections remain a major concern in terms of morbidity and mortality among dialysis patients. Various hospital cohorts and studies indicate that the risk of TB in haemodialysis and peritoneal dialysis patients is 3 to 25 times higher than in the general population, mainly due to the impaired cellular immunity associated with renal failure [6,7]. In this context of immunosuppression, TB is particularly frequent, atypical and severe [8].

Iliac bone TB remains a rare manifestation of extra-pulmonary TB. Its diagnosis can be challenging due to its similarity to other bone conditions.

This case underscores the importance of early consideration of TB in the differential diagnosis of bone disorders, particularly in endemic regions. Classic symptoms such as local pain, fever and emaciation can be confusing, but as in the present case study, osteoarticular TB can manifest as cold abscesses containing serum, leukocytes, caseous material, bone debris and Koch’s bacillus (BK). These abscesses follow a variety of routes, crossing the periosteum, ligaments and fascia planes [9]. TB must, therefore, be strongly suspected in the presence of soft-tissue abscesses associated with joint pain, particularly in our patient.

Biological diagnosis of extra-pulmonary forms of TB is often complex. Indeed, in these forms, the lesions are generally paucibacillary and the sites from which biological samples are taken are often very difficult to access [10,11]. In this case study, iliac bone puncture was not feasible. We, therefore, opted for the puncture of a nearby soft tissue collection.

The diagnosis of bone TB is guided by non-specific biological parameters, such as CRP and procalcitonin, in combination with radiological images (CT and MRI). It is then confirmed by specific microbiological examinations, involving conventional techniques (DE and culture) and molecular biology.

DE allows for the detection of the BK after preparation of the samples with Ziehl–Neelsen staining, enabling a rapid diagnosis within 2 h. Although inexpensive and highly sensitive for bacillary forms, DE remains less informative in extra-pulmonary TB. Its sensitivity is 70%, but its specificity remains very low [12].

The fluorescence microscope allows for the detection of BK after staining with auramine. This method, less expensive, has an improved sensitivity of 84%, with a specificity of 97% [13]

Culture is the reference method for diagnosing TB, whether pulmonary or extra-pulmonary. Its sensitivity ranges from 60% to 90%, with a specificity of 100%. This method can be used to diagnose microscopy-negative forms of TB, in particular extra-pulmonary TB, which is often difficult to diagnose by DE. Culture allows for the performance of an antibiogram. LJ medium is the most commonly used. The time required for colony growth is significantly extended, ranging from 3 to 4 weeks, and up to 6 weeks in paucibacillary forms [12]. The prolonged bacterial growth time can delay the initiation of anti-TB treatment, thereby unfavourably impacting the prognosis.

The advent of molecular biology has revolutionized the diagnosis of TB, especially with the approval in 2010 by the WHO of the Xpert/MTB/Rif test or GeneXpert^®^ [14]. This tool has significantly improved sensitivity and reduced the time required to confirm TB to less than 2 h. For the diagnosis of extra-pulmonary TB, it stands out with a sensitivity of 77.3% and a specificity of 98.2% [12]. In addition to detecting the DNA of the MTBC, GeneXpert^®^ also allows for the detection of rifampicin resistance. However, it is important to note that a negative GeneXpert^®^ result does not rule out TB.

## Conclusion

Primary iliac bone TB remains a rare clinical entity, even in endemic regions. It requires a rigorous approach combining clinical evaluation, advanced medical imaging and microbiological analyses to achieve an accurate diagnosis. Recent advances in molecular biology, in particular with the advent of the GeneXpert®, have considerably improved patient management, providing microbiological evidence in less than 2 h, thereby allowing early initiation of anti-TB treatment. Analysing the journey that concluded with the diagnosis of primary bone TB in our patient, we were able to highlight the challenges encountered in the early diagnosis of this condition, often masked by atypical clinical presentations.

## Abbreviations

BK: Koch’s bacillus
CRP: C-reactive protein
CT: computed tomography
DE: direct examination
LJ: Lowenstein–Jensen
MRI: magnetic resonance imaging
MTBC: *Mycobacterium tuberculosis* complex
PCR: polymerase chain reaction
TB: tuberculosis
WHO: World Health Organization
